# The first space-filling polyhedrons of polymer cubic cells originated from Weaire-Phelan structure created by polymerization induced phase separation

**DOI:** 10.1038/s41598-022-22058-7

**Published:** 2022-11-09

**Authors:** Naofumi Naga, Masumi Jinno, Yuting Wang, Tamaki Nakano

**Affiliations:** 1 Department of Applied Chemistry, College of Engineering, Shibaura Institute of Technology, 3-7-5 Toyosu, Koto-ku, Tokyo, 135-8548 Japan; 2 Graduate School of Engineering and Science, Shibaura Institute of Technology, 3-7-5 Toyosu, Koto-ku, Tokyo, 135-8548 Japan; 3 Institute for Catalysis and Graduate School of Chemical Sciences and Engineering, Hokkaido University, N 21, W 10, Kita-ku Sapporo, 001-0021 Japan; 4 Integrated Research Consortium On Chemical Sciences, Institute for Catalysis, Hokkaido University, N 21, W 10, Kita-ku Sapporo, 001-0021 Japan

**Keywords:** Chemistry, Materials science

## Abstract

The Weaire–Phelan structure is a three-dimensional structure composed of two different polyhedra having the same volume, i.e., pyritohedron and truncated hexagonal trapezohedron. It was proposed by Weaire and Phelan in 1993 as a solution of the Kelvin problem of filling space with no gaps with cells of minimum surface area and equal volume. It was found in physical systems including liquid foam and a metal alloy while it has never been constructed as organic materials. We report herewith the first polymeric Weaire–Phelan structure constructed through phase-separation of a single polymer species that is synthesized by simple polyaddition between tetrakis(3-mercaptopropionate) and 1,6-diisocyanatohexane. The structure has the order of micrometers and is amorphous unlike reported crystal structures similar to the Weaire–Phelan structure.

**“**Tessellation of space into cells of equal volume with the least surface area” has been referred to as the Kelvin problem from the nineteenth century. In 1887, Lord Kelvin proposed a convex uniform honeycomb formed by a bi-truncated octahedron^1^. This form is called “Kelvin structure” and has been widely believed as the most efficient form in tessellation^2^. After more than 100 years later, Weaire and Phelan discovered a more efficient form, so called “the Weaire-Phelan structure”, by computer simulations^3^. The Weaire-Phelan structure is composed by two kinds of cells with equal volume. One is a tetrakaidecahedron with two hexagonal and twelve pentagonal phases, and the other is an irregular dodecahedron with pentagonal faces. The arrangement of 3/4 of the tetrakaidecahedron cells and 1/4 of the dodecahedron cells in a specific way forms the Weaire-Phelan structure.

The family of the Weaire-Phelan structure is summarized in Fig. 1. The Weaire-Phelan structure inspired the architecture design of Beijing National Aquatics Center, called ‘Water Cube’, for the 2008 Summer Olympics^4^. The Weaire-Phelan structure has been found in physical materials only in two cases, i.e., liquid foam with the order of millimeters in size made from a detergent solution^5,6^ and a Pd-Pb alloy^7^. However, it has never been constructed for organic materials including polymer, especially, single-component polymeric material. The highly ordered three-dimensional structure in polymeric materials could open a way to novel function. In this work, we successfully constructed a Weaire-Phelan structure using a network polythiourethane through polymerization-induced phase separation forming closely packed uniform particles of the order of micrometer for the first time. The polymer was synthesized by simple polyaddition reaction between tetrakis(3-mercaptopropionate) (PEMP), a multi-functional primary thiol compound as a “joint” source monomer, and hexamethylene diisocyanate (HDI), a diisocyanate as a “linker” source monomer, in the presence of triethyl amine (TEA) as a base catalyst in toluene (Fig. 2a).

**Figure 1:**
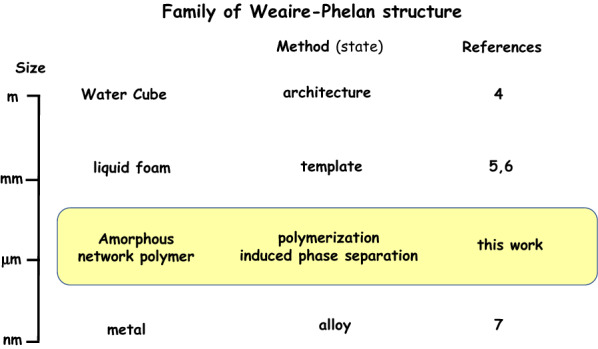
Family of Weaire-Phelan structure.

**Figure 2:**
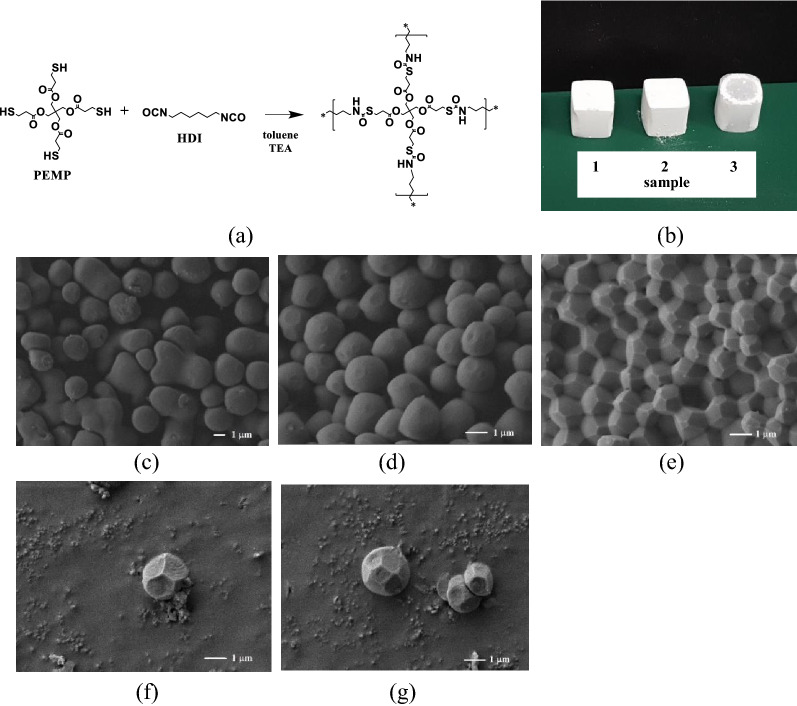
(**a**) Synthetic scheme of network polymers through the addition reaction of PEMP and HDI in toluene, and photos and images of the polymers, (**b**) Photos of PEMP-HDI network polymers from the reaction systems at monomer concentrations in the reaction systems of sample 1 (left): 25 wt%, sample 2 (center): 30 wt%, and sample 3 (right): 35 wt%, SEM images of PEMP-HDI network polymers (**c**) 25 wt% (sample 1), (**d**) 30 wt% (sample 2), and (**e**)–(**g**) 35 wt% (sample 3).

Crystal or mesophase structures with some similarity to the Weaire-Phelan structure and with sizes of tens of nanometers are known, for examples, Type I clathrate structure for alkali metal silicides and germanides^8^ and for gas hydrates composed of methane, propane and carbon dioxide^9^, and a Frank-Kasper phase (FK A15) of some self-assembled organic molecules such as dendritic liquid crystals, block copolymers, and giant surfactants^10–15^. However, organic amorphous materials having the Weaire-Phelan structure are unprecedented. The polymer Weaire-Phelan structure we report here is characterized by amorphousness and the size of the order of micrometers prepared by very facile synthetic procedures.

The synthetic methodology employed in this work is based on the “joint-and-linker” concept. The addition reaction between a multi-functional monomer as a source of “joint” (“joint”-source monomer) and α,ω-bifunctional monomer as a source of “linker” (“linker-source monomer) forms polymer network^16–24^. The joint-and-linker synthesis so far reported often preferentially yielded porous polymers by polymerization-induced phase separation via spinodal decomposition. When the porous structure is fixed at an early stage of spinodal decomposition, a monolithic structure composed of co-continuous structure of polymer backbone and vacant space is produced. While isolated particles are generated at a later stage, and aggregated particles are formed upon an increase in the polymerization rate. The size and morphology of porous structure are decided by the ratio of the polymerization (network formation) rate to the phase separation rate. The relative ratio can be controlled by designing monomers and catalysts to tune reactivity and by considering miscibility between the polymer network and the solvent^25–34^.

The addition reaction between PEMP and HDI was conducted at monomer concentrations of 25, 30, and 35 wt%, {(PEMP + HDI)/(PEMP + HDI + Toluene)}*100 = 25, 30, and 35 wt% ([PEMP] = 0.28, 0.34, and 0.40 M), resulting in samples 1, 2, and 3, respectively, in toluene in the presence of TEA as catalyst at room temperature at a feed ratio of [PEMP]/[HDI] = 1/2 ([–NCO] = [–SH]) and at a ratio of [TEA]/[–SH] = 1/100. All the reaction mixtures quickly turned opaque within a few minutes of the catalyst addition and yielded network polymers as shown in Fig. 2b. In the FT-IR spectra of the PEMP-HDI network polymer, the absorption peak based on –NCO group at 2250 cm^-1^ almost completely disappeared and a peak based on thiol-urethane bond emerged at 3300 cm^-1^ (Fig. S1), supporting the intended reaction mechanism. Wide-angle x-ray diffraction patterns of the polymers showed only broad halo profiles, revealing that the polymers are amorphous.

The scanning electron microscopy (SEM) images of the PEMP-HDI network polymers are shown in Figs. 2c–e. The polymer obtained at a monomer concentration of 25 wt% (sample 1) showed a morphology composed of particles with spherical shapes and fused spherical shapes with a diameter of about 1.5 µm (Fig. 2c). The reaction at a monomer concentration of 30 wt% also produced spherical particles with slightly larger diameters than those in sample 1 (sample 2, Fig. 2 d) where the particles appeared more densely accumulated than those in sample 1. It is noteworthy that the surface of the particles sample 2 were slightly truncated. On the other hand, the sample obtained at a monomer concentration of 35 wt% (sample 3) clearly exhibited space-filling polyhedron particles with pentagon and hexagon faces on the surface which may correspond to the polyhedrons the Weaire-Phelan structure. Isolated particles of the PEMP-HDI network polymer of sample 3 appeared distorted tetrakaidecahedron and dodecahedron units of the Weaire-Phelan structure, in (Figs. 2 e,f,g and Fig. 3). The bulk density of the PEMP-HDI network polymer increased with an increase in the monomer concentration of the reaction system, i.e., 0.808 g/cm^3^ (sample 1), 0.861 g/cm^3^ (sample 2), and 1.094 g/cm^3^ (sample 3), respectively.

**Figure 3:**
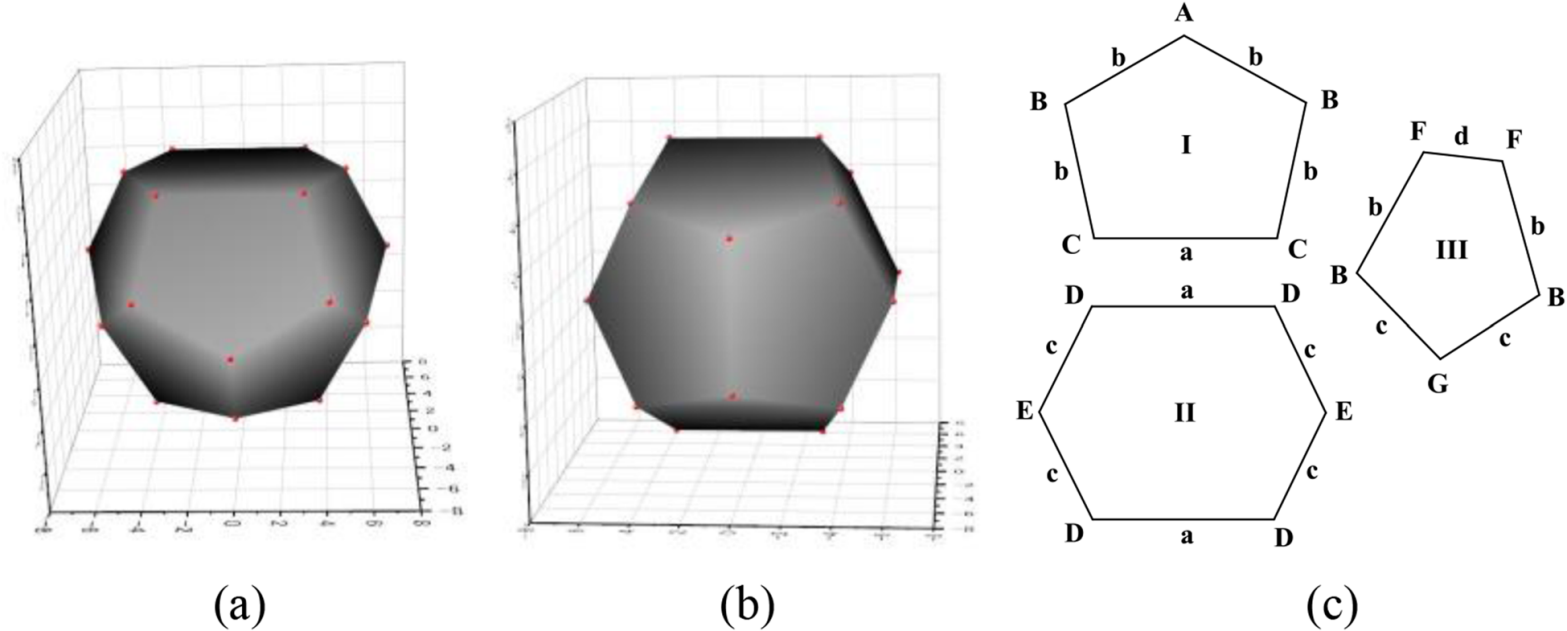
(**a**) Tetrakaidecahedron, (**b**) dodecahedron polyhedrons, and (**c**) pentagon and hexagon structures in the theoretical Weaire-Phelan structure.

The polyhedron structure of sample 3 was further studied by 3D SEM in more detail (Fig. 4). Space-filling polyhedrons with structures exactly matching those of the polyhedrons of the Weaire-Phelan structure were clearly detected. Figure 4b shows the plots of the structures reproduced based on the three-dimensional coordinates of the vertices of particles, observed in the rectangular frame of the 3D SEM image (Fig. 4a) of the surface of the PEMP-HDI polymer sample 3. The bird's eye views of PEMP-HDI polymer clearly indicated that the three-dimensional polyhedron particles with micrometer-order sizes were formed over the surface of sample 3.

**Figure 4:**
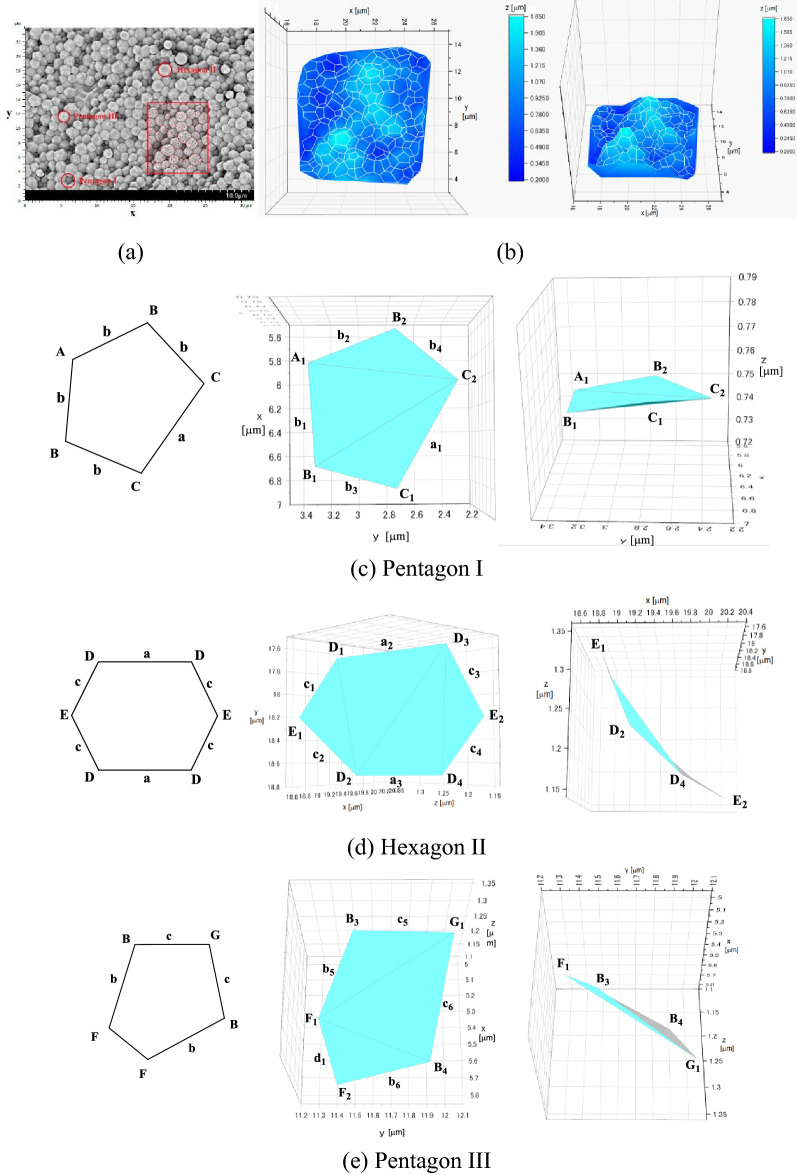
(**a**) A 3D SEM image, (**b**) bird’s eye views of the PEMP-HDI polymer of sample 3, 3D plots of (**c**) Pentagon I, (**d**) Hexagon II, and (**e**) Pentagon III.

The theoretical Weaire-Phelan structure without any anisotropy is composed of two pentagons and one hexagon structures as shown in Fig. 3c. From this view, the structures of the polyhedrons observed on the surface of sample 3 were quantitatively examined. Figures 4c–e compare the pentagon structure I, hexagon structure II, and pentagon structure III extracted from the 3D SEM image as marked in red in Fig. 4a with the corresponding theoretical structures in Fig. 3c. The side lengths and angles of the polygons in Fig. 4 were calculated from the coordinate points in the 3D SEM image, which are available in supporting information. The numerical data of the side lengths and angles of the polygons from the observed structures and those from the theoretical structures are summarized in Table. 1.

**Table 1:** Structure comparison of polyhedrons observed in 3D SEM with theoretical structure in the Weaire-Phelan structure.

Sides & angles ^a^	Side length µm	Length ratio ^b^	Theoretical length ratio	Angle º	Theoretical angle º	Error %
**Pentagon I**						
a_1_ (µm)	0.92	1.07a’	1a			+ 7.1
b_1_ (µm)	0.79	0.92a’	0.76a			+ 21.0
b_2_ (µm)	0.64	0.75a’	0.76a			− 1.3
b_3_ (µm)	0.55	0.64a’	0.76a			− 15.8
b_4_ (µm)	0.57	0.66a’	0.76a			− 13.2
A_1_ (º)				110.5	121.6	− 9.1
B_1_ (º)				113.0	106.6	+ 6.0
B_2_ (º)				110.4	106.6	+ 3.6
C_1_ (º)				105.7	102.6	+ 3.0
C_2_ (º)				100.3	102.6	− 2.2
**Hexagon II**						
a_2_ (µm)	0.80	0.89a’’	1a			− 11.0
a_3_ (µm)	0.82	0.91a’’	1a			− 8.8
c_1_ (µm)	0.54	0.60a’’	0.66a			− 9.1
c_2_ (µm)	0.59	0.66a’’	0.66a			± 0.0
c_3_ (µm)	0.78	0.87a’’	0.66a			+ 31.4
c_4_ (µm)	0.64	0.71a’’	0.66a			+ 7.6
D_1_ (º)				119.5	116.6	+ 2.5
D_2_ (º)				121.4	116.6	+ 4.1
D_3_ (º)				122.5	116.6	+ 5.1
D_4_ (º)				128.8	116.6	+ 10.5
E_1_ (º)				125.7	126.9	− 1.0
E_2_ (º)				101.4	126.9	− 20.0
**Pentagon III**						
b_5_ (µm)	0.43	0.79b’	1b			− 21.3
b_6_ (µm)	0.51	0.93b’	1b			− 6.7
c_5_ (µm)	0.53	0.97b’	0.86b			+ 12.8
c_6_ (µm)	0.53	0.97b’	0.86b			+ 12.8
d_1_ (µm)	0.35	0.64b’	0.58b			+ 10.3
B_3_ (º)				101.5	106.6	− 4.8
B_4_ (º)				107.0	106.6	+ 0.4
F_1_ (º)				135.3	112.2	+ 20.6
F_2_ (º)				101.4	112.2	− 9.6

a: Coordinate points are available in supporting information, b: a_1_ + b_1_ + b_2_ + b_3_ + b_4_ = 4.04a’, b_5_ + b_6_ + c_5_ + c_6_ + d_1_ = 4.3b’, a_2_ + a_3_ + c_1_ + c_2_ + c_3_ + c_4_ = 4.64a’’.

As for pentagon I (Fig. 4c and Table. 1 (i)), the observed angles were in good agreement with the theoretical values while the ratios of the observed side lengths slightly deviated from those of the theoretical lengths. A relatively large deviation was detected in a side length (c_3_) in hexagon II (Fig. 4 d and Table. 1 (ii)), and the other the side lengths and all angles showed relatively good agreement between the observed and theoretical values. The structure of hexagon II appears very close to that of the corresponding theoretical structure. Pentagon III structure fairly well coincided with the theoretical structure with at most 20% errors both in the side lengths and angles (Fig. 4 e and Table. 1 (iii)).

The formation process of the space-filling polyhedrons of the PEMP-HDI network polymer is proposed as follows. The porous structures in samples 1 and 2 were formed by the phase separation induced by the polyaddition between PEMP and HDI in toluene. Thereafter, two types of phase separation process are possible, *i.e.,* nucleation growth and spinodal decomposition as shown in Fig. 5. Although, monolithic structure was not detected in the SEM images of the present research (Figs. 2 c,d), phase separation should preferentially occur via spinodal decomposition, as previously observed in other porous polymers prepared by the “joint-and-linker” method^21,23^.In the SEM image of sample 1 (Fig. 2c), half-fused particles were detected which should be formed via spinodal decomposition. At an early stage of spinodal decomposition, co-continuous monolithic structure should be formed, and further phase separation transforms the monolithic structure to the structure with dispersed droplet-type morphology by interfacial tension, which forms the connected particles. The formation of the half-fused particles in sample 1 (Fig. 2c should be completed at this transition state of the phase separation^35–37^.A further progress of the phase separation forms isolated spheres accompanied by their growth. The surface morphology in sample 2 should be completed at this stage, as shown in Fig. 2d. An increase in monomer concentration in the present reaction systems would increase the phase separation rate, which should finalize the phase-separated structure at later stages of phase separation. As described above, the small circle planes, which were derived from the truncated structures, were detected on the surface of the particles in sample 2 (Fig. 2d). Contact between particles may possibly play a key role in the formation of the polyhedral shapes. An increase in monomer concentration in the reaction increased the space occupancy in the porous polymer by the polymer network, leading to the slightly more transparent appearance of sample 3 than samples 1 and 2 (Fig. 2b). The polyhedrons observed in sample 3 (Fig. 2e) can be formed by 3D occupation of vacant space in the porous polymer by the spheres. The polyhedrons may be formed on an increase in the number of particles per unit space at the highest monomer concentration used in this work where the spheres changed into the polyhedrons in order to facilitate the densest (most dense) packing in space without leaving any gaps. In this process, truncation seems to be indispensable in reducing gaps between particles, and the spheres eventually took the shapes of tetrakaidecahedron and dodecahedron which can completely fill up the space without any gaps left.

**Figure 5:**
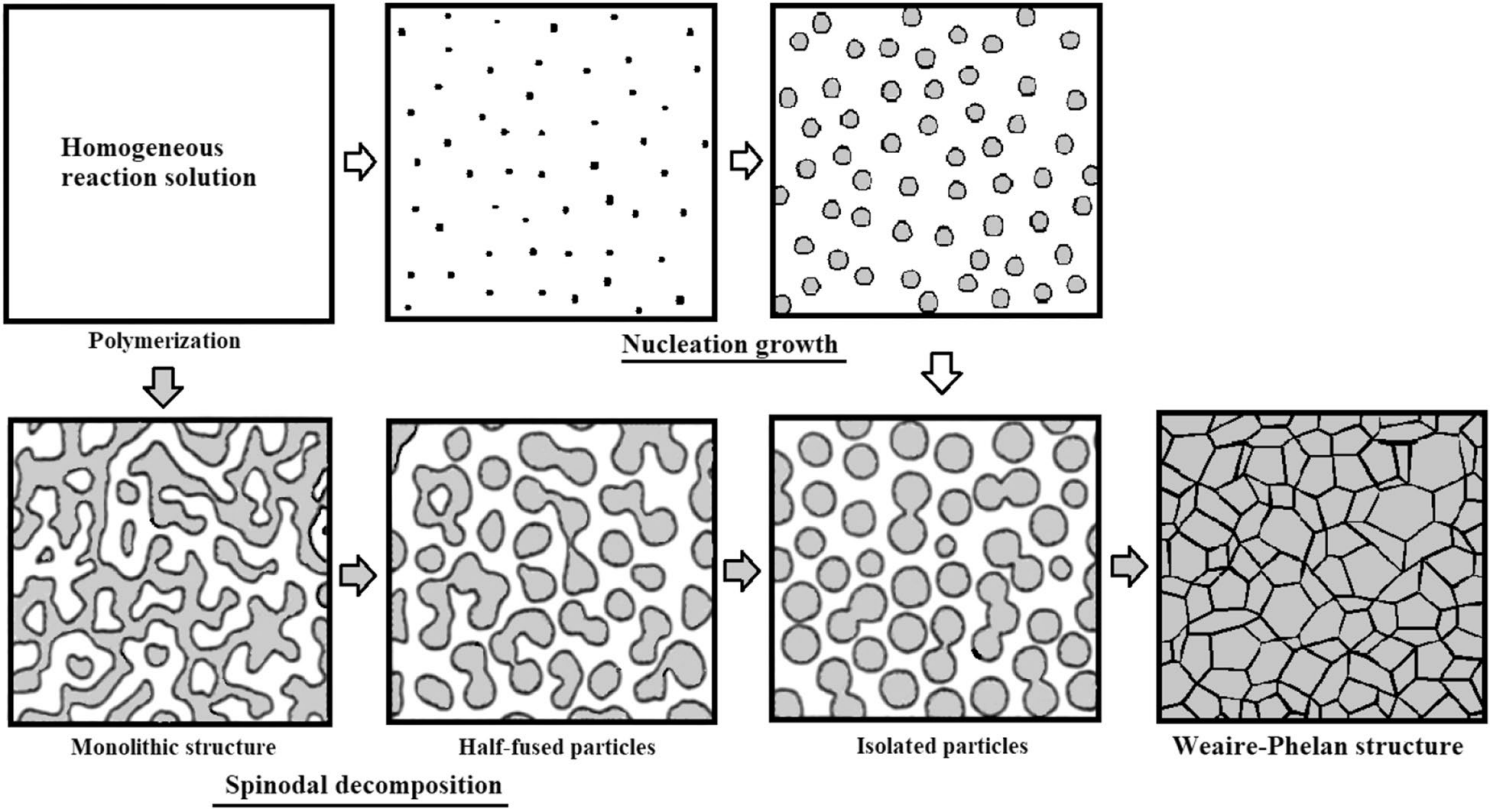
Phase separation models via nucleation growth and Spinodal decomposition to form Weaire-Phelan structure.

The cubic honeycomb proposed by Kelvin was thus formed for the Weaire-Phelan structure in this work. This is the first example of Weaire-Phelan structure made from a polymer species and of solid-polymer cubic honeycomb created by polymerization-induced phase separation. The material synthesized in this work may be potentially applicable as photonic^38–40^, separation, catalytic, nano-medical and structural materials based on the new synthetic methodologies and structural concepts.

While there still are slight deviations in specification between the theoretical polyhedrons of the perfect Weaire-Phelan structure, further investigations are ongoing targeting to minimize the deviations. Control of space filling and tessellation should be useful not only from a basic view but also from views of development of advanced materials with unforeseen functions.

## Supplementary Information

Supplementary Information.

## Data Availability

Data generated and analyzed during this study are provided as source data with this paper or included in the Supplementary Information. Further data are available from the corresponding authors on reasonable request.
