# Supplementary material for: The first space-filling polyhedrons of polymer cubic cells originated from Weaire-Phelan structure created by polymerization induced phase separation
Source: Sci Rep. 2022 Nov 9;12:19141. doi: 10.1038/s41598-022-22058-7 (PMC9646714; doi:10.1038/s41598-022-22058-7)
Supplement: Supplementary file 1 — Supplementary Information. [file 41598_2022_22058_MOESM1_ESM.docx]

Supplementary information

**The first space-filling polyhedrons of polymer cubic cells originated from Weaire-Phelan structure created by polymerization induced phase separation**

Naofumi Naga, Masumi Jinno, Yuting Wang, Tamaki Nakano

Correspondence to: [nnaga@sic.shibaura-it.ac.jp](mailto:nnaga@sic.shibaura-it.ac.jp), [tamaki.nakano@cat.hokudai.ac.jp](mailto:tamaki.nakano@cat.hokudai.ac.jp)

Materials and Methods

**Methods**

**Materials.** Pentaerythritol tetrakis(3-mercaptopropionate) (PEMP) (SC Organic Chemical Co., Ltd.), hexamethylene diisocyanate (HDI, Tokyo Chemical Industry Co., Ltd.), triethylamine (TEA, Kanto Chemical Co., Inc.), and toluene (Kanto Chemical Co., Inc.) were commercially obtained, and used without further purification.

**Synthesis of porous polymers.** Addition reaction of PEMP with HDI in 25 wt% of monomer concentration (sample 1) is described as an example. PEMP (0.549 g, 1.124 mmol), toluene (3.21 mL), and TEA (6.3 μL, 45.0 μmol) were added to a 10 mL ampoule tube and stirred by vortex mixer. HDI (0.36 mL, 2.248 mmol) was added to the reaction system and stirred by vortex mixer for a few seconds immediately and stayed at room temperature. The reaction system turned white in 5min and was stored for 24 h at room temperature to complete the reaction. The porous polymer was immersed in plenty methanol to wash and replace the solvent for 24 h. The obtained porous polymer was dried under the atmospheric conditions for 24 h and in vacuo at room temperature for 6 h. The porous polymers with different monomer concentration were prepared with the same procedure.

**Analytical Procedures.** FT-IR spectra of the reaction solutions and polymer networks were recorded on a Jasco FT/IR-410 (JASCO Corporation). A reaction solution without TEA was prepared between KBr-Real Crystal IR-Card and Slip (International Crystal Laboratories). A sample of the porous polymer was prepared in KBr disk. Thirty scans were accumulated from 4000 to 500 cm^-1^.

Scanning electron microscopy (SEM) images of the porous polymers were observed by a JEOL JSM-7610F microscope with an LEI detector at an acceleration voltage of 3.0 kV. 3D SEM image of a polymer, Pt coated sample 3, were recorded by Hitachi High-Tech Corporation SU3800 at an acceleration voltage of 5.0 kV. The 3D structure was analyzed using the Hitachi map 3D software (https://www.hitachi-hightech.com/global/science/products/microscopes/electron-microscope/tabletop-microscopes/map3d/).

**Fig. S1.** FT-IR spectra of (a) PEMP-HDI/toluene mixture before the reaction at a monomer concentration of 25 wt%, and (b) the resulting PEMP-HDI network polymer (sample 1).

**Table S1.** Coordinate points of Pentagon I (Fig. 4 (c)).

| μm | A_1_ | B_1_ | B_2_ | C_1_ | C_2_ |
| --- | --- | --- | --- | --- | --- |
| x | 5.87 | 6.66 | 5.61 | 6.82 | 6.00 |
| y | 3.33 | 3.28 | 2.75 | 2.75 | 2.33 |
| z | 0.749 | 0.740 | 0.751 | 0.761 | 0.748 |

**Table S2.** Coordinate points of Hexagon II (Fig. 4 (d)).

| μm | D_1_ | D_2_ | D_3_ | D_4_ | E_1_ | E_2_ |
| --- | --- | --- | --- | --- | --- | --- |
| x | 18.9 | 19.0 | 19.7 | 19.8 | 18.7 | 20.2 |
| y | 17.7 | 18.7 | 17.6 | 18.7 | 18.2 | 18.2 |
| z | 1.30 | 1.27 | 1.17 | 1.19 | 1.36 | 1.15 |

**Table S3.** Coordinate points of Pentagon III (Fig. 4 (e)).

| μm | B_3_ | B_4_ | F_1_ | F_2_ | G_1_ |
| --- | --- | --- | --- | --- | --- |
| x | 5.05 | 5.72 | 5.42 | 5.75 | 5.22 |
| y | 11.5 | 11.9 | 11.3 | 11.4 | 12.0 |
| z | 1.26 | 1.21 | 1.16 | 1.11 | 1.35 |
